# Survey on large language model annotation of cellular senescence from figures in review articles

**DOI:** 10.1186/s44342-024-00011-6

**Published:** 2024-06-17

**Authors:** Yuki Yamagata, Ryota Yamada

**Affiliations:** 1 R-IH, BioResource Research Center RIKEN, Tsukuba, 305-0074 Japan; 2 BioResource Research Center RIKEN, Tsukuba, 305-0074 Japan; 3 Fuku Inc, Tokyo, 113-0033 Japan

**Keywords:** Data curation, Cellular senescence, LLM

## Abstract

This study evaluated large language models (LLMs), particularly the GPT-4 with vision (GPT-4 V) and GPT-4 Turbo, for annotating biomedical figures, focusing on cellular senescence. We assessed the ability of LLMs to categorize and annotate complex biomedical images to enhance their accuracy and efficiency. Our experiments employed prompt engineering with figures from review articles, achieving more than 70% accuracy for label extraction and approximately 80% accuracy for node-type classification. Challenges were noted in the correct annotation of the relationship between directionality and inhibitory processes, which were exacerbated as the number of nodes increased. Using figure legends was a more precise identification of sources and targets than using captions, but sometimes lacked pathway details. This study underscores the potential of LLMs in decoding biological mechanisms from text and outlines avenues for improving inhibitory relationship representations in biomedical informatics.

## Introduction

Chronic diseases associated with aging pose a significant challenge to an aging society [1]. We developed a Homeostasis Imbalance Process Ontology (HoIP) [2] based on manual annotation to elucidate the fundamental mechanisms, particularly cellular senescence. However, innovative solutions are required to streamline this process in terms of scalability and facilitation of updates. In response to these challenges, this study investigates the efficacy of large language models (LLMs) using the GPT-4 with vision (GPT-4 V) and GPT-4 Turbo (gpt4-1106-preview) [3] for annotating figures present in review articles.

This study aimed to explore the capabilities of LLMs in the systematic categorization of various biomedical factors and in annotating the directionality of causal relationships and regulatory mechanisms, with a particular focus on cellular senescence. Our approach contributes to streamlining the annotation process, enhancing the accuracy and efficiency of the data, and clarifying how LLMs interpret data in the study of aging and cellular senescence.

## Methods

**Fig. 1:**
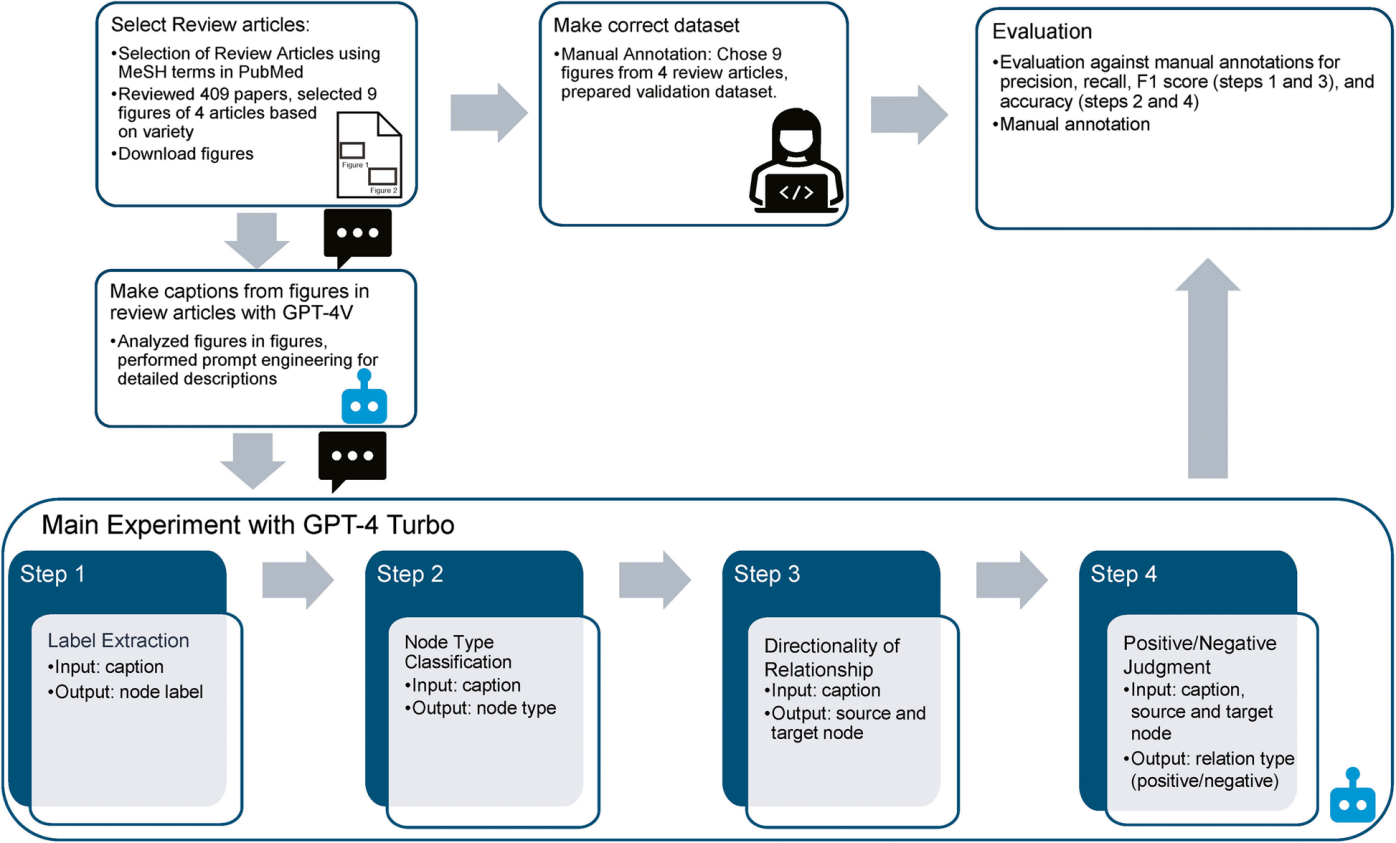
Overview of the large language model (LLM) annotation process for figures in review articles. Our LLM annotation task is based on the captions from figures in cellular senescence review articles created by GPT-4 V. Subsequently, the main experiment consists of four steps: label extraction, node-type identification, directionality, and relationship analysis using GPT-4 Turbo

This study explored annotations using LLMs in figures from review articles depicting cellular senescence, from basic biology to medical domains involving diverse molecules and processes. Figure 1 presents an overview of the LLM annotation process used in this study. The PubMed database was searched for open-source review articles with downloadable figures. After reviewing 409 articles, 4 were selected [1, 4–6] based on their diverse contents and number of references. From these studies, we selected nine manually annotated figures to prepare the dataset for validation. We conducted zero-shot learning experiments using OpenAI’s GPT-4 V API to investigate the ability of LLMs to explain the factors related to cellular senescence, as depicted in the figures. Next, we performed prompt engineering to generate detailed descriptions (captions) of cellular senescence. After converting all figures into captions, the main experiments were performed using GPT-4 Turbo (gpt-4–1106-preview). All experiments were performed five times. Initially, the labels for entities associated with cellular senescence were extracted. Subsequently, the nodes were classified into five categories relevant to the biological phenomena: molecules, compounds, cells, processes, and diseases. Furthermore, nodes with identified sources and targets, along with positive/negative regulation, were annotated to investigate their ability to understand the mechanisms. The performance of the LLM annotations was evaluated against a dataset of manual annotations using precision, recall, and F1 score metrics for Steps 1 and 3, and accuracy for Steps 2 and 4.

## Results and discussion

**Fig. 2:**
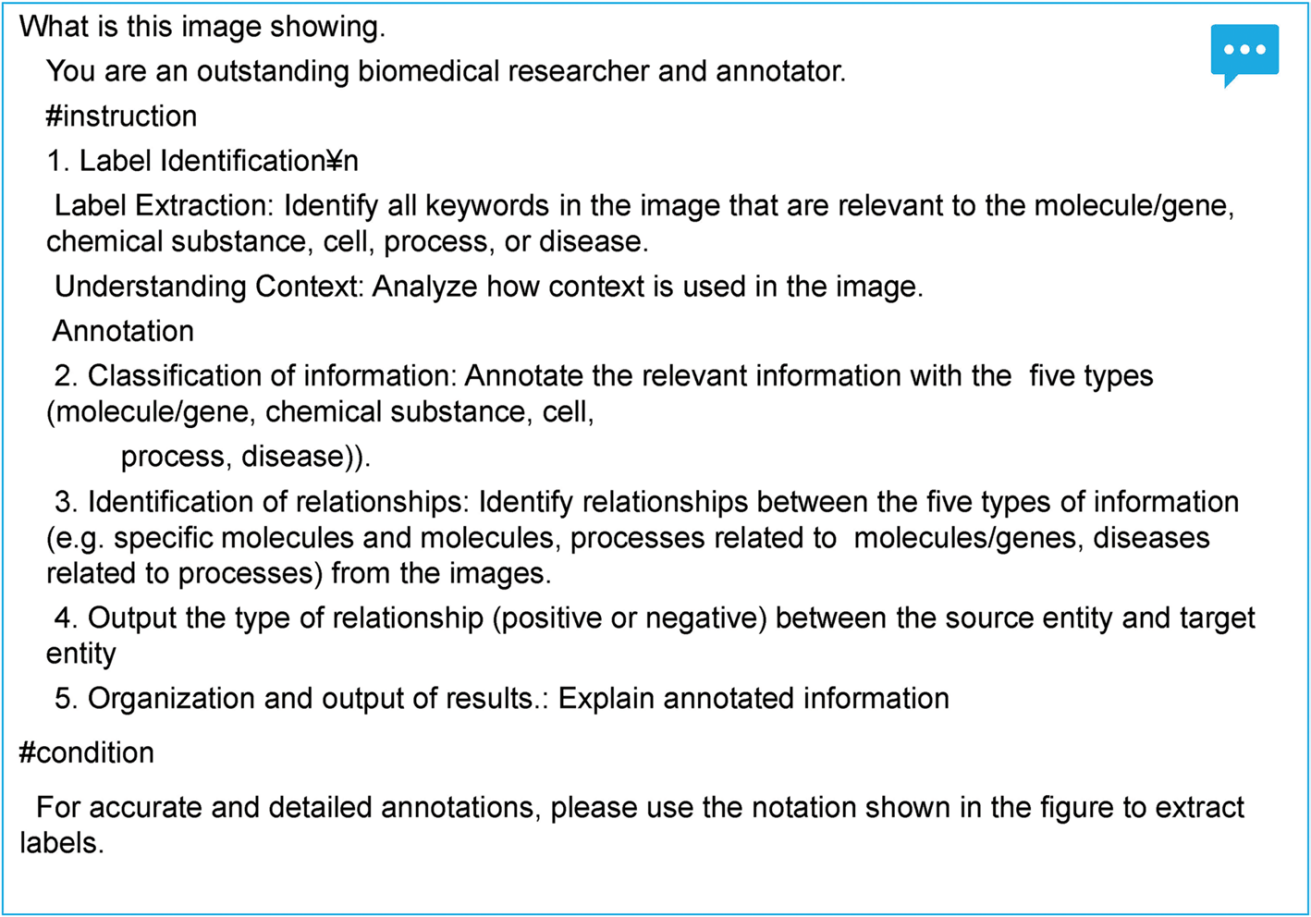
Prompt engineering example for review article figures. This figure illustrates the prompt engineering approach used to improve the annotation capabilities of large language models (LLMs)

**Fig. 3:**
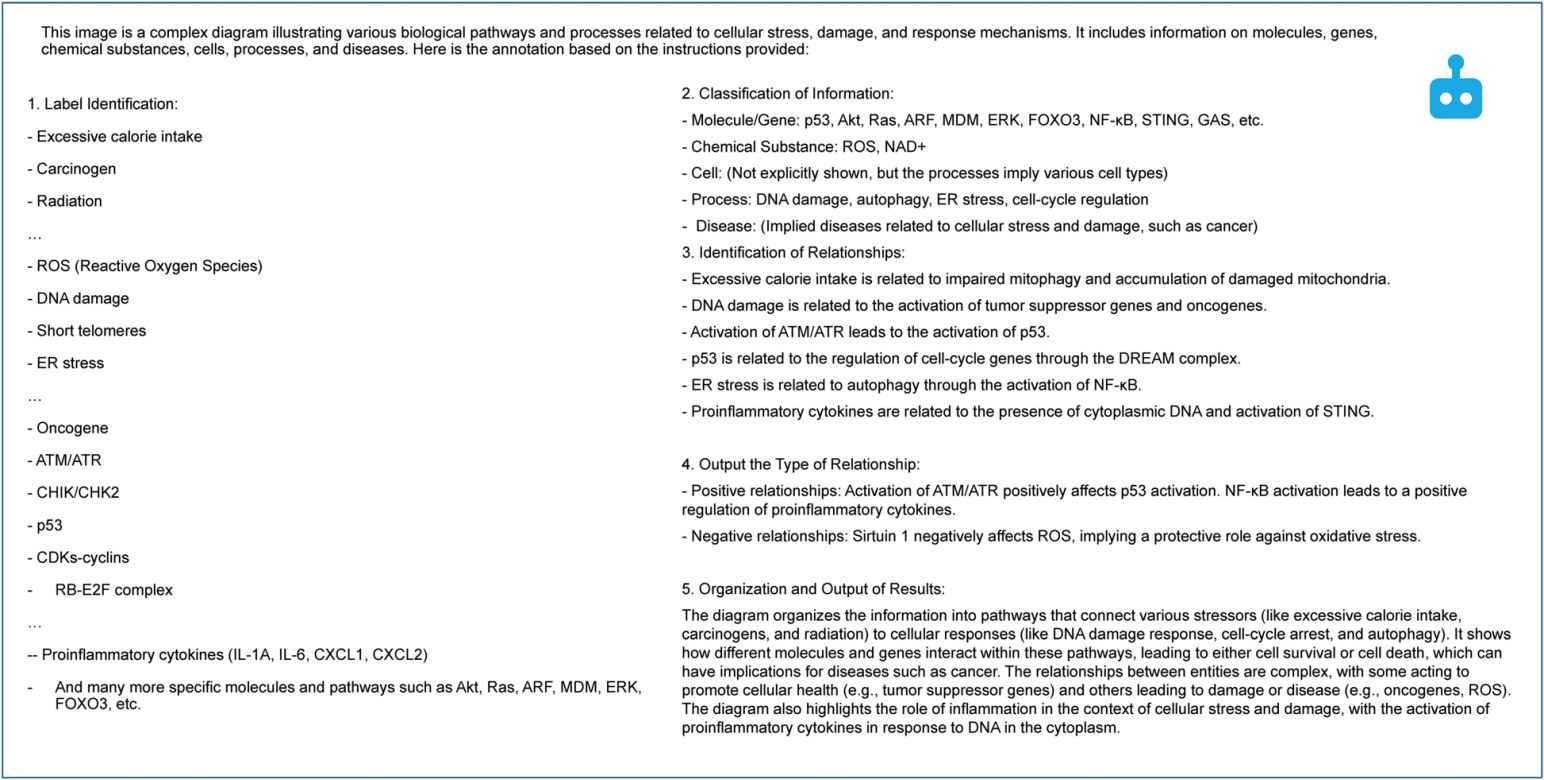
Caption generation example for a review article figure. This figure demonstrates an example of captions created by large language models (LLMs) from Fig. 2 of one review article [1]

In this study, node-type classification refers to node characterization into five distinct categories. These categories included molecules, compounds, cells, processes, and diseases. Further, relation directionality involves identifying the direction of upstream or downstream networks, specifying which nodes serve as sources, and which are targets. Positive/negative identification refers to the regulation of the process between the identified sources and targets; positive (promoting or activating) or negative (inhibiting). In preliminary experiments using GPT-4 V, we tested the ability of LLMs to decipher molecules and cellular senescence mechanisms using zero-shot learning. These trials indicated the proficiency of LLMs in identifying well-known molecules and outlining senescence-related processes. Nonetheless, the models struggled with the figure elements, particularly the arrows. By refining our prompts (Fig. 2), we improved the generation of detailed descriptions and converted images into text (captions) for further annotation (Fig. 3). Therefore, we have set the stage for more advanced annotation tasks. Tables 1 and 2 present an overview of experimental results. The experiments demonstrated that the LLMs effectively extracted labels from the figures, achieving a precision of 0.76, recall of 0.69, and F-measure of 0.72. Several of the identified errors were attributed to compound words. The node-type identification experiment yielded an accuracy of > 80%, indicating the capability of correctly categorizing entities. When classifying entities, errors were observed when categorizing molecules and processes. Cytokines such as IL-6 and IL-8 were mistakenly identified as processes, whereas complexes such as CDK cyclins were incorrectly classified as processes. Furthermore, at times, “senescence” was erroneously annotated as a cell, possibly due to the frequent descriptions of “senescence cell” in biomedical articles, which may have influenced the LLM’s learning. In addition, entities related to inflammation, which should have been correctly categorized as pathological processes, were misclassified as diseases. Thus, these results highlight the challenges faced by LLM in accurately distinguishing closely related biological entities, underscoring the need for further refinement of their classification tasks.

**Table 1:** Step-wise performance of large language model (LLM) annotation metrics. Precision, recall, and F1 score for Steps 1 and 3, focusing on label identification and relation directionality

Step	Precision	Recall	F1 score
Step 1: Label identification	0.76	0.69	0.72
Step 3: Relation directionality	0.46	0.28	0.34
Step 3: Relation directionality (*figures with* < *30 node*s)	0.72	0.57	0.64

**Table 2:** Step-wise performance of large language model (LLM) annotation metrics. Accuracy for binary classifications in Step 2 and Step 4, assessing correct identifications of node types and regulation types, simplified by specific types

Step	Accuracy
Step 2: Node-type classification	0.84
Process	0.83
Molecule	0.84
Compound	0.57
Cell	1.0
Disease	0.98
Step 4: positive/negative identification	0.82
Positive regulation	0.85
Negative regulation	0.66

While annotating relation directionality, it was observed that the performance of the LLMs in identifying relationships decreased as the number of nodes increased. Across all the diagrams, the average metrics were as follows: precision, 0.46; recall, 0.28; and F1 score, 0.34. However, upon excluding diagrams containing > 30 relationships, the performance metrics were notably improved: precision, 0.72; recall, 0.57; and F1 score, 0.64. The initial annotations across all diagrams indicated a lower recall, which may be attributed to the decreased ability of the models to correctly identify relevant relationships in diagrams with a higher density of nodes.

Notably, incorrect interpretations, such as the inversion of source and target between “Senescence” and “Chronic Inflammation,” occurred even with a small number of nodes. This may be attributed to the LLM’s reliance on extensive background knowledge rather than the figure itself, as evidenced by the LLM-generated caption derived from the figure stating, “Chronic inflammation can promote senescence through the release of inflammatory cytokines.”

To further evaluate the annotation capabilities of LLMs, we conducted additional experiments to assess their ability to recognize and annotate complex biological relationships in the figure legends. We used figure legends instead of captions, for example, “Senescence can, in turn, drive the consequential aging hallmarks in response to damage: stem cell exhaustion and chronic inflammation.” Consequently, identification of sources and targets of the nodes in the figure were accurately identified using the LLM. Furthermore, for diagrams with many nodes, we observed that recognizing the direction of the source–target relationship can be significantly improved by utilizing the detailed description in the legends, which serve as viable substitutes for captions. However, many legends frequently oversimplify or neglect the intermediate relationships described in the diagrams. In particular, the legends tend to directly link well-established molecules and processes. Consequently, although the legends effectively summarize the figure, they may fall short of exploring the mechanisms, particularly when detailed pathways are absent.

While annotating relationships, a significant challenge has emerged in accurately identifying inhibitory relationships, often symbolized by “-|” in biomedical diagrams. This difficulty in translating the visual representations of inhibition from figures into precise annotations underscores the broad issue of interpreting the complicated mechanisms across our experiments. The statistical analysis highlighted a notable disparity in accuracy between positive and negative relationships. The mean positive accuracy exceeds 80%, significantly surpassing the mean negative accuracy of < 70%. To explore this further, we conducted an additional experiment with ChatGPT, focusing on understanding its complex pathways, including multiple inhibition relationships. The core question was whether the meaning of inhibition is fully understood. To address this issue, we provided textual explanations within our prompts, clarifying that the inhibition of an inhibitory factor logically results in its promotion. The results revealed that LLMs understand that inhibiting an inhibitory relationship acts as a promoter and that pathways with multiple inhibitory relationships can be explained consistently. This finding indicates that despite the initial difficulties with direct recognition from figures, LLMs can interpret complex relationships through textual information, thereby demonstrating their potential to interpret intricate biological mechanisms.

Our study investigated another aspect of the LLMs’ ability to infer relevant background knowledge, which was not explicitly mentioned in the figures. In one diagram, only the direct relationships between molecules such as p16, p21, p53, p14, and p27 and growth arrest are depicted. Interestingly, LLM expanded on this by stating: "Negative relationships: The presence of p16, p21, p53, p15, p14, and p27 reinforces growth arrest, preventing cell proliferation." Although this interpretation deviates slightly from the explicit content of the diagrams, it serves as a helpful contribution to the exploration of cellular senescence mechanisms, illustrating the ability of the LLM to infer beyond the immediate data presented, such as human experts in the domain, thereby clarifying the possible mechanisms.

## Conclusion

In summary, this study offers an in-depth analysis of entity classification, directional annotation, and positive/negative regulation annotation within cellular senescence mechanisms through multiple experimental trials. Although LLMs exhibit promising capabilities in certain aspects of classification and annotation, to improve classification task accuracy in LLMs, especially in distinguishing between categories such as processes and molecules, an embedding technique should be explored. Such an embedding technique may significantly reduce categorical ambiguities when discerning differences between terms used in articles concerning immunological and inflammatory contexts, where, specifically, cytokines such as IL-6 and IL-8 are extensively discussed. The accurate identification and annotation of inhibitory relationships remains a significant challenge. Future research should focus on enhancing the ability of models to accurately interpret and integrate complex biomedical knowledge, with particular emphasis on the precise representation of inhibitory relationships in biological processes. Ontologies have emerged as promising strategies to address these challenges. For this strategy, we used the Monarch Initiative plugin [7] for ChatGPT to annotate the Protein Ontology [8] terms of 13 well-known molecules. These results yielded annotations for various species. In the following prompt, specifying the requirements for human molecules, the LLM correctly annotated 11 out of the 13 molecules. These discrepancies pertain to species mismatch and entirely different molecular identifications. We will further scrutinize the remaining molecules to refine the annotations. In the future, we intend to leverage LLMs to reference these existing ontologies and enhance the accuracy and depth of the annotations. Future research should improve the accuracy and refine classification tasks using ontology hierarchies. Gradually, the understanding and classification accuracy of the LLM will be refined by generalizing entities, that is, superclasses, and subsequently classifying subclass entities into additional categories. This approach underscores the potential of integrating LLMs with structured biomedical knowledge using ontologies to improve the understanding and representation of complex biological phenomena.

## Data Availability

The data described in this article is available from https://github.com/yuki-yamagata/LLM_annotation_cellular_senescence
